# A New Approach for Indexing Honey for Its Heath/Medicinal Benefits: Visualization of the Concept by Indexing Based on Antioxidant and Antibacterial Activities

**DOI:** 10.3390/medicines5040135

**Published:** 2018-12-17

**Authors:** Mahmud Masalha, Saleh Abu-Lafi, Basheer Abu-Farich, Mahmoud Rayan, Nael Issa, Mouhammad Zeidan, Anwar Rayan

**Affiliations:** 1 Laboratory of Microbiology, QRC-Qasemi Research Center, Al-Qasemi Academic College, P.O. Box 124, Baka EL-Garbiah 30100, Israel; mahmudmasalha@gmail.com; 2 Faculty of Pharmacy, Al-Quds University, Abu-Dies 144, Palestine; sabulafi@staff.alquds.edu; 3 QRC-Qasemi Research Center, Al-Qasemi Academic College, P.O. Box 124, Baka EL-Garbiah 30100, Israel; af_basheer@qsm.ac.il; 4 Drug Discovery Informatics Lab, QRC-Qasemi Research Center, Al-Qasemi Academic College, Baka El-Garbiah 30100, Israel; Mahmoud_ryan@hotmail.com; 5 Science Education Department, Al-Qasemi Academic College, P.O. Box 124, Baka EL-Garbiah 30100, Israel; nael-e@qsm.ac.il; 6 Molecular Genetics and Virology Laboratory, QRC-Qasemi Research Center, Al-Qasemi Academic College, P.O. Box 124, Baka EL-Garbiah 30100, Israel; mouhammad.zeidan7@gmail.com; 7 Institute of Applied Research-Galilee Society, Shefa-Amr 20200, Israel

**Keywords:** honey, antibacterial, antifungal, antioxidant, free radical scavenging, health benefits’ index

## Abstract

**Background:** The goals of the current study were to address a new concept termed a health benefits’ index (HBI) and to verify the type of correlation between the pricing of honey and its HBI/medicinal properties. Diverse types of honey from different origins and places were investigated for their antioxidant and antimicrobial activity. **Methods:** We have utilized a modified protocol of the DPPH assay for measuring free radical scavenging and the microdilution test for the determination of antibacterial/antifungal minimum inhibitory concentrations (MICs). MICs were determined against *Staphylococcus aureus*, *Escherichia coli*, *Salmonella typhimurium*, and *Candida albicans* microorganisms. Employing a “combined benefits approach” enabled us to attach to each honey type a unique number of HBI that correlate with honey health and medicinal values. **Results:** The various types of honey demonstrated significant but variable antioxidant, antibacterial, and antifungal activities. Types of wildflower-labeled honey were found to have a wide range of HBI values and medicinal properties, probably due to their containing different nectar contents/phytochemicals. Moreover, an inconsiderable correlation was detected between the market prices of different types of honey and their HBIs. **Conclusions:** The proposed index of health benefits could be recalculated/updated following measurement of more and more medicinal properties, such as anti-inflammatory, antidiabetic, and anticancer activities. This index could be used as an effective tool for consumers of honey to evaluate the real value of the purchased product.

## 1. Introduction

Honey is a natural product that is produced by honeybees from the nectar of flowers [1]. It has nutritional, cosmetic, and therapeutic value [2,3], and it is considered one of the most important natural products that has been used by humankind since ancient times [4,5]. Currently, general magazines, newspapers, leaflets of natural products, and scientific journals publish a great amount of information on the utility of honey for controlling many human diseases and refer to a wide variety of mysterious properties [6,7], ranging from anti-aging to fertility. Much evidence indicates that honey can exert numerous health beneficial effects, including wound healing [8]; antioxidant [9]; anti-inflammatory [10,11], anticancer [12], antimicrobial [13], and antidiabetic [14,15] effects; and respiratory [16], gastrointestinal [17,18], cardiovascular [19], neurological [20], and nervous system [21] protective effects. Honey could exert its potential therapeutic effects in the treatment of diseases through the combination of a wide range of active compounds. Natural products have been optimized to interact with biological targets via a long natural selection process [22], and consequently, nature is rich in bioactive ingredients [23,24,25] and has been considered the best source of medicines for millennia [26].

Today, about three hundred types of floral honey have been documented [27]. This variety is linked to a wide diversity in the types of nectar collected by honeybees. Honey is composed of about 83% solids; the rest is water. The main portion of the solid fraction consists of 95 to 97% carbohydrates. Moreover, honey contains proteins (diastase, invertases, glucose oxidase, catalase, and acid phosphatase) [28], vitamins, amino acids (all of the nine essential amino acids and all of the nonessential amino acids, except asparagine and glutamine) [29], minerals (phosphorus, sodium, calcium, potassium, sulfur, magnesium, chlorine) [30], and other organic acids [31,32], such as flavonoids, polyphenols, alkaloids, glycosides, anthraquinone, and volatile compounds [33,34,35,36]. 

Scientific researchers have identified more than six hundred volatile chemicals that may contribute to honey’s health benefits [37]. The fraction of volatile chemicals in honey is low, but consist of aldehydes, ketones, alcohols, acids, esters, hydrocarbons, derivatives of benzene, derivatives of terpene, norisoprenoids, and cyclic compounds [36,38]. Some bioactive chemicals, such as quercetin, luteolin, galangin, isorhamnetin, and kaempferol, exist in almost all types of honey [39,40]. Flavonoids and polyphenols, which act as antioxidants, are the two main bioactive groups of chemicals that present in honey. Recent studies have shown the existence in honey of approximately thirty kinds of polyphenols [20,41]. Their levels can fluctuate significantly, depending on the source of the nectar and on the environmental conditions. As well, the major phenolic and flavonoid chemicals in honey are composed of gallic acid, caffeic acid, ferulic acid, cinnamic acid, chlorogenic acid, syringic acid, coumaric acid, ellagic acid, benzoic acid, hesperetin, galangin, myricetin, apigenin, quercetin, isorhamnetin, chrysin, naringenin, catechin, luteolin, *p*-coumaric, and kaempferol [42,43,44]. The aforementioned components are the mostly responsible ingredients for the antioxidant, antimicrobial, anti-inflammatory, anticancer, and antidiabetic effects of honey.

Although a significant number of papers have been published so far concerning the chemical components and health benefits of certain honeys, to our knowledge, no equation was proposed for indexing the various types of honey for their health benefits values. This issue instigated this research study.

## 2. Materials and Methods

### 2.1. Honey Sample

Honeys were purchased in closed cans from several vendors and stored unopened at room temperature in a dry place. It is worth mentioning that the honey sources were indicated, but not the names of the companies producing the honey, so that the scientific article would not be an advertisement. 

### 2.2. Chemicals and Standards

The chemicals gallic acid, nystatin, tetracycline, 2,2-Diphenyl-1-picrylhydrazyl ([DPPH), *p*-iodonitrotetrazolium chloride, kanamycin, and the solvents (ethanol and DMSO) were purchased from Sigma Aldrich, Rehovot, Israel. The four standard microbial strains used here were *Staphylococcus aureus* strain SH1000, *Escherichia coli* strain 8739, *Salmonella typhimurium* strain LT2, and *Candida albicans* strain 10231. All of the aforementioned microbial strains were purchased from the American Type Culture Collection (ATCC), Manassas, VA, USA.

### 2.3. Free Radical Scavenging Activity

The free radical scavenging of the different kinds of honey was conducted by microdilution DPPH assay, with some modifications. The assay was performed using two-fold serial dilution in pasteurized water. The tests were carried out in 96-well, flat-bottomed micro-titration plates. To 0.1 milliliter of the honey solutions, 0.1 milliliter of ethanolic DPPH solution (100 ppm) was added. The final concentrations of the honey solutions were (w/w%): 16.67%, 8.33%, 4.17%, 2.08%, 1.04%, 0.52%, 0.26%, 0.13%, 0.065%, 0.033%, and 0.016%. The mixture was shaken and allowed to stand for 30 min in the dark at room temperature. The absorbance of the solution was measured at 620 nm and converted into a percentage of free radical scavenging using the following equation:

Free radical scavenging% = 100*{1 − [(A_sample_ − A_blank_1_))/(A_control_ − A_blank_2_)]}

where A_sample_ is the absorbance of the honey and DPPH mixture solution, A_blank___1_ is the absorbance of the honey solution, A_control_ is the absorbance of the ethanolic solution of DPPH, and A_blank___2_ is the absorbance of ethanol.

Gallic acid was used as a positive control. The free radical scavenging was expressed in terms of the EC_50_ (the amount of antioxidant necessary to decrease the initial DPPH absorbance by 50%). The EC_50_ value for each type of honey was determined by extracting the value from the equation for the linear part of the graph. We substituted 50% for the *y* value while calculating the concentration value of the *x*-axis. 

### 2.4. Antibacterial and Antifungal Activities

The micro-dilution test was used to determine the minimum inhibitory concentrations (MICs) of the different honey samples. A broth micro-dilution assay was performed using two-fold serial dilution in brain heart infusion (BHI) broth. The test was carried out in 96-well, flat-bottomed micro-titration plates. The cell suspension was prepared in BHI broth with an optical density equivalent to the 0.5 McFarland standard and diluted 1:100 in BHI broth to obtain a final concentration of 5 × 10^5^ clone-forming units per milliliter (CFU/mL). Controls of broth only and broth with bacteria without any of the antibacterial agents were also included in each plate. One hundred µl of antibacterial agent was put in the first microplate well and serially diluted in BHI broth. One hundred µl, corresponding to 5 × 10^5^ CFU/mL, was added to all of the wells. The plates were incubated at 37 °C for 18 h overnight. Tetracycline was used as a positive control for *S. aureus* and *E. coli*, while nystatin was used as positive control for *Candida albicans* and *Salmonella typhimurium* strain LT2. The minimum inhibitory concentration (MIC) was defined as the lowest concentration able to inhibit the visible growth of bacteria in the triplicate wells. After visual determination of the MIC, twenty microliters of *p*-iodonitrotetrazolium violet (8 mg/mL EtOH) were added to each well. The plate was further incubated for 30 min and assessed visually for any change in color from yellow to pink, which would indicate reduction of the dye due to bacterial growth.

### 2.5. Indexing Method

Employing a “combined benefits approach” enables the attachment to each honey type a health benefits’ index (HBI) that correlates with honey health and medicinal values. The HBI concept is based on the assumption that honey type, which possesses more health and medicinal benefits, is high-valued. As well, the necessity by the community for producing an index that quantifies the overall health and medicinal quality of each type of honey is the basis for proposing the construction of HBI index.

Equation (1)

(1) $$HBI=\sqrt[{m}]{\prod_{i=1}^{n}\delta_{i}\;\left(\frac{E^{i}}{E^{r}}\right)}$$

where:

*n* is the number of health/medicinal properties that are used for construction of HBI index. 

*δ_i_* refers to the contribution factor of the indicated health/medicinal property *i*. The value of *δ_i_* might range between 0 and 1 and equal 1 (one) if all health and medicinal benefits are proposed to contribute equally to the index.

*E^i^* is the efficient concentration toward the indicated health/medicinal property *i* (the used parameters are EC_50_, MIC, etc.).

*E^r^* is the efficient concentration toward the health/medicinal property of the reference honey (*Rhamnus* honey type from Yemen as proposed in the current study).

*m* is the total contributions and it is calculated according to equation II:

Equation (2)

(2) $$m=\overset{n}{\underset{i=1}{\sum }}\delta_{i}$$

According to equation I, *HBI* value equal 1.0 means similar health and medicinal benefits as exerted by *Rhamnus* honey type from Yemen (reference honey). A value of *HBI* less than 1.0 means higher health and medicinal benefits, while a value of *HBI* greater than 1.0 means less health and medicinal benefits than the reference honey. 

The equivalent dose (*E_d_*) is the indicated dose of the reference honey (*Rhamnus* honey type from Yemen, in this case) multiplied by *HBI.*

### 2.6. Statistical Analysis

All experiments were carried out in quadruplicate, unless otherwise indicated, and all statistical analyses were conducted using Excel spreadsheet software (v16.0, Microsoft, Redmond, WA, USA). The data are expressed in terms of the average ± standard deviation. Differences among the groups were evaluated by applying one-way analysis of variance (ANOVA). The quality of correlation between any two parameters was evaluated based on the value of the coefficient of determination (*R*^2^). Reliability decreases with a decrease in the *R*^2^ value (*R*^2^ > 0.85 means very good, 0.7 > *R*^2^ < 0.85 means reliable, while less than 0.7 means less-reliable). A *p*-value of less than 0.05 was considered statistically significant.

## 3. Results and Discussion

Over the years, some honeys have acquired an appealing reputation for curing certain ailments. For example, Yemeni Mountain *Sidr* honey has been well known for its health benefits in the Middle East for centuries. It is a unifloral honey that made by bees fed on flower nectar from the *Ziziphus spina-christi* tree, which is known also as the Christ’s thorn jujube [7]. The Arabs believe Yemeni Mountain Sidr honey is superior to high-priced Manuka honey from New Zealand, which has garnered more publicity in the West. In the literature, there is still no solid answer to what makes certain types of honey better than others. Today, the market is full of adulterated artificial honeys claimed to be natural. This study, therefore, aimed to address a new method for indexing honey for its medicinal benefits and investigate correlations between the price of honey and its index (potential medicinal benefits). The selected honeys, which included types of honey from Israel, Palestine, and Bulgaria, were tested against some pathogenic microorganisms. The effectiveness of the MIC of different antimicrobial agents, namely, tetracycline and tetracycline nystatin, was tested against *Staphylococcus aureus, Escherichia coli, Salmonella typhimurium*, and *Candida albicans* microorganisms (Table 1). All the types investigated exhibited significant but variable antioxidant and antimicrobial activity. No activity was detected in any of types against the *Salmonella typhimurium* and *Candida albicans* strains, even at the highest concentration of 16.67% (w/w%).

Twenty honey types were examined, of which only four were classified as high-priced (Table 2); the rest were moderately or low-priced. The results shown in Table 2 for the MIC values with respect to *E. coli* and *Salmonella* species of the high-priced *Rhamnus* (*sidr*) and *Tamarix* (*saal haar*) types, in comparison to the moderately priced citrus and wild flowers, were very similar, but they were much lower than those of their positive controls, tetracycline and kanamycin, respectively. Moreover, the antioxidant activities of the high-priced types (*Rhamnus* and *Acacia tortilix*) showed half EC_50_ values, indicating superior antioxidant effects compared to those of the cheaper honeys from citrus and wild flowers.

**Figure 1:**
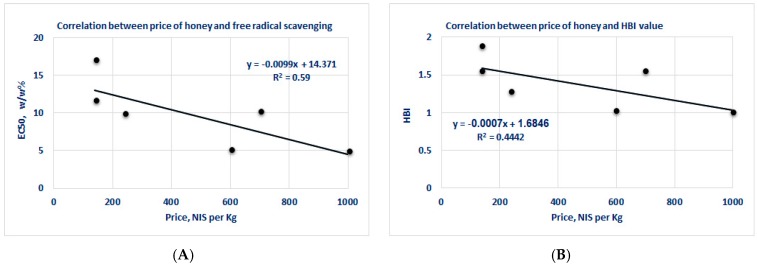
Correlation between the price of honey and free radical scavenging (**A**, left side) as well as prices and HBI values (**B**, right side), as detailed in Table 2.

The correlations between the honey prices and the EC_50_ for antioxidant activity or HBI values are depicted in Figure 1. The closely scattered EC_50_/HBI values make it hard to draw a decisive distinction based on prices. Therefore, an inconsiderable correlation was detected between the prices of the different kinds of honey and their antioxidant activity or HBI. Table 3 shows five different types of European honey in a similar price range. Their antioxidant and antimicrobial values are close, with only insignificant differences. 

Table 4 presents the EC_50_ and MIC values with respect to *E. coli* and *Salmonella* species of four local, commercial, and relatively low-priced wildflower honeys that are produced by different vendors from Israel. The four types of honey inhibited the growth of *E. coli* and *Salmonella* to almost the same extent; however, an inconsiderable correlation was detected between the prices of the different kinds of honeys and their antioxidant activity.

Table 5 represents the free radical scavenging capabilities of the same types of wildflower honey, but from different batches. The average free radical scavenging EC_50_ of the four batches is 8.7 w/w%, while the standard deviation is 5.1 w/w%, indicating relatively wide spectrum of free radical scavenging capacities for wildflower types of honey.

No activity was detected for any of the honey types against the *Salmonella typhimurium* and *Candida albicans* strains, even at the highest concentration of 16.67% (w/w%). 

It is worth mentioning that all types of honey, which are labeled as “wildflower”, were found to be very diverse in their biological activities and health benefits’ indexes (HBIs). This could be explained by the claim that each type of wildflower-labeled honey contains different nectar contents/phytochemicals.

## 4. Conclusions

It is reported in the scientific literature that there are large variations in the ingredients of honey, which may be due to spatial and temporal variations in the sources of nectar. As a consequence, variations in their biological effects and health benefits can be expected. Thus, an evaluation of the biomedical benefits of different honey types may provide valuable information on their quality and their possible therapeutic potential for treating several health disorders in humans, and can determine if variations in pricing are justified and whether there are correlations between prices and health benefits. In this study, we investigated the medicinal properties of the different honeys, with an emphasis on their antioxidant, antibacterial, and antifungal activities. All of the honey types that were tested herein exhibited significant but varied antioxidant, antibacterial, and antifungal activities. No correlation was detected between the price of a particular type of honey and its antioxidant, antibacterial, and antifungal activities. All types of wildflower-labeled honey were found diverse in health benefits’ indexes (HBIs) and medicinal properties, probably due to their containing different nectar contents/phytochemicals. 

The proposed index of health benefits could be recalculated following measurement of more and more medicinal properties, such as anti-inflammatory, antidiabetic, and anticancer activity. This could be used as an effective tool for consumers of honey to appreciate the real value of the purchased product.

## Figures and Tables

**Table 1:** Minimum inhibitory concentration (MIC) values of the antimicrobial agents used.

Pathogenic Microorganisms	Antimicrobial Agent	MIC, μg/mL
*Staphylococcus aureus*	Tetracycline	0.09
*Escherichia coli*	Tetracycline	3.12
*Salmonella typhimurium*	Kanamycin	10.0
*Candida albicans*	Nystatin	1.55

**Table 2:** Commercial honey types from Palestine, * prices, and antioxidant and antimicrobial activity. Each number is an average of four replications. *HBI* was calculated according to equation 1 based on both properties (antioxidant and antimicrobial activities).

Kind of Honey (Origin)	Price ($)	EC_50_, (w/w%)	MIC (*E. coli*)	MIC (*Salmonella*)	*HBI*
*Rhamnus* (Yemen, termed *sidr*)	270	4.8	0.52	0.52	1.00
*Tamarix* (Yemen, termed *saal-haar*)	189	7.7	1.04	0.52	1.55
*Acacia tortilis* (Yemen, termed *somar*)	162	5.0	0.52	0.52	1.02
*Rhamnus* (Jericho, Palestine)	65	7.8	0.52	0.52	1.27
*Citrus* (Jericho, Palestine)	38	17.0	0.52	0.52	1.88
*Wild flowers* (Jericho, Palestine)	38	0.116	0.52	0.52	1.55

* Purchased from MamLakat Al-Asal (“kingdom of honey”) shop, Nablus, Palestine.

**Table 3:** Commercial Bulgarian honey types, prices, and antioxidant and antimicrobial activity.

Kind of Honey	Price ($)	EC_50_, (w/w%)	MIC (*E. coli*)	MIC (*Salmonella*)	*HBI*
*Silybum* (*milk thistle*)	6.8	10.05	0.26	0.52	1.18
*multicolored herbal honey*	6.8	12.1	0.52	0.52	1.59
*Linden honey*	6.8	12.66	-	0.52	1.62
*Wildflower*	6.8	20.1	0.26	0.26	1.45
*Acacia*	6.8	10.1	0.52	0.52	1.45

**Table 4:** Commercial Israeli honey types, prices, and antioxidant and antimicrobial activity.

Kind of Honey	Price ($)	EC_50_, (w/w%)	MIC (*E. coli*)	MIC (*Salmonella*)	*HBI*
Wildflower (vendor 1)	13.5	9.4	0.52	0.52	1.40
Wildflower (vendor 2)	27	16.34	1.04	1.04	2.61
Wildflower (vendor 3)	13.5	6.8	0.52	0.52	1.19
Wildflower (vendor 4)	13.5	9.2	0.52	0.52	1.38

**Table 5:** Free radical scavenging values for the wildflower honeys (from different bottles that were purchased from the same vendor, [Israeli honey factory, termed above as vendor 3]).

Kind of Honey	Price ($)	EC_50_, (w/w%)
Wildflower	12	6.78
Wildflower	12	4.8
Wildflower	12	5.8
Wildflower	12	17.5
	Average	8.7
	STDEV	5.1
